# Characterization and Bone Response of Carbonate-Containing Apatite-Coated Titanium Implants Using an Aqueous Spray Coating

**DOI:** 10.3390/ma10121416

**Published:** 2017-12-11

**Authors:** Ryo Yagi, Chihiro Mochizuki, Mitsunobu Sato, Takeshi Toyama, Masatsugu Hirota, Tohru Hayakawa, Chikahiro Ohkubo

**Affiliations:** 1 Department of Removable Prosthodontics, Tsurumi University School of Dental Medicine, 2-1-3, Tsurumi, Yokohama, Kanagawa 230-8501, Japan; okubo-c@tsurumi-u.ac.jp; 2 Division of Liberal Arts, Center for Promotion of High Education, Kogakuin University, 2665-1, Nakano, Hachioji, Tokyo 192-0015, Japan; mochizukic@cc.kogakuin.ac.jp; 3 Department of Applied Physics, School of Advanced Engineering, Kogakuin University, 2665-1, Nakano, Hachioji, Tokyo 192-0015, Japan; lccsato@cc.kogakuin.ac.jp; 4 Department of Materials and Applied Chemistry, College of Science and Technology, Nihon University, 1-8-14, Surugadai, Kanda, Chiyoda, Tokyo 101-8308, Japan; touyama.takeshi@nihon-u.ac.jp; 5 Department of Dental Engineering, Tsurumi University School of Dental Medicine, 2-1-3, Tsurumi, Yokohama, Kanagawa 230-8501, Japan; hirota-masatsugu@tsurumi-u.ac.jp (M.H.); hayakawa-t@tsurumi-u.ac.jp (T.H.)

**Keywords:** carbonate-containing apatite, titanium, aqueous spray coating, dental implant

## Abstract

We performed thin carbonate-containing apatite (CA) coating on titanium (Ti) by an aqueous spray coating (ASC) method that consisted of a Ca-CO_3_-PO_4_ complex. Two different CA coatings were produced by two different spray amounts and were heat-treated after spraying. We evaluated three-dimensional structures, adhesiveness to Ti, and durability of the CA film. In addition, we performed immersion experiments in simulated body fluid (SBF), and bone responses were evaluated after implantation into a femoral bone defect in rats. The bonding ability of ASC-coated implant into the bone was examined by push-in tests. Unique network structures with small particles were identified on CA coatings. Although heat treatment produced no significant difference in surface morphology, scratch tests revealed that heat treatment improved the adhesion of CA coatings to Ti. Crystal formation progressed on CA-coated specimens, and the sample placement direction influenced crystal formation and growth in SBF immersion. Animal implantation experiments revealed significantly greater bone-to-implant contact ratio and bone mass in both cortical and bone marrow, respectively, four weeks after implantation. Push-in tests suggested that the bonding of the CA coating to Ti is clinically acceptable. Therefore, we conclude that CA coating to Ti by the ASC method would be possible for clinical applications, including dentistry.

## 1. Introduction

Due to its superior mechanical properties, corrosion resistance, and biocompatibility, titanium (Ti) is widely used in the fields of dentistry, orthopedic surgery—Such as for artificial joints and stents, and maxillofacial surgery [1,2]. In addition to the tight and direct bonding of Ti (known as osseointegration) [3], a type of calcium phosphate coating on Ti, known as hydroxyapatite coating, improves and accelerates the bone healing process [4,5,6]. Various methods for hydroxyapatite coating have been reported, including plasma spray [6,7,8], the sol–gel method [9,10], physical vapor deposition (PVD) [11,12,13], and electrostatic spray deposition (ESD) [14,15]. Among them, PVD can produce thin and uniform apatite coatings, and ESD can deposit carbonate-containing apatite (CA) films on a Ti substrate, which have chemical components that resemble bone minerals [16].

Regarding the effectiveness of plasma spray coating, Oirschot et al. reported that plasma spray hydroxyapatite coatings with high roughness significantly improve the osteophilic capacity of titanium surfaces using a goat transverse model in lumbar spine [17]. Clinical assessments of hydroxyapatite-coated dental implant for one year confirmed the achievement of osseointegration by observing computed tomography. Hydroxyapatite coating was applied by the arc micro plasma spray technique [18]. On the contrary, a systematic review of the long-term success of calcium phosphate plasma spray-coated dental implants in clinical trials with at least five years of follow-up suggested that long-term cumulative success rate demonstrated very weak evidence for progressive complications around calcium phosphate plasma spray-coated dental implants [19].

The effectiveness of thin hydroxyapatite coating formed by the PVD method has also been reported. Magnetron sputtering or the pulsed laser deposition technique is commonly employed in the PVD method. It is reported that osteoblasts cultured on the crystalline apatite surface produced by PVD were more active [20]. Madi et al. [21] evaluated the soft and hard tissue responses to experimental peri-implantitis of the mandibles of beagle dogs around hydroxyapatite-coated implants and found that thin sputter hydroxyapatite-coated implants possess favorable osteoconductivity without exacerbating further peri-implant tissue breakdown during the progression of peri-implantitis. It is also reported that thin sputter hydroxyapatite-coated implants enhanced the remodeling activity in osteoporotic ovariectomized rat [22].

Previously, we developed a new coating procedure known as the molecular precursor method [23,24]. In this method, a molecular precursor solution, which is an ethanol solution containing calcium-EDTA (ethylenediamine-tetraacetic acid )complexes and phosphate salts (with a Ca/P ratio of 1.67), is applied onto a Ti surface. Following heating the Ti substrate at 600 °C for 2 h, a thin CA film can be deposited on the Ti surface. Thin CA coatings produced by the molecular precursor solution showed greater bone-to-implant contact following implantation into the femoral condyles of rabbits [25]. Moreover, the molecular precursor method was effective for producing thin CA coatings on partially stabilized zirconia [26,27]. However, in this method, ethanol was used as a solvent, and Ti was heated for 2 h.

More recently, Mochizuki et al. [28] developed a new coating method that used an aqueous spray solution and was referred to as the aqueous spray coating (ASC) method. A stable aqueous solution consisting of calcium hydroxide, CO_2_, and phosphoric acid (Ca/P ratio was adjusted to 1.67) was used for spray coating. A stable Ca–CO_3_–PO_4_ complex was formed in the aqueous solution. This method is simple, the CA thin film is free of volatile organic compounds (VOCs), and the unique network structure of CA film could be deposited on the Ti substrate. The chemical structure of the resulting CA film is CA_10_(PO_4_)_5_(HPO_4_)_5_(CO_3_)(OH)·CO_2_. The ASC method produced several rounded and small particles on the CA films, and previous cell assays using osteoblast-like cells reported that the obtained network size and border height in the network structure on the CA films enhanced the initial cell attachment, cell proliferation, and expression level of osteocalcin [29].

In the present study, we characterized, in more detail, the thin CA films using the ASC method. In particular, we examined three-dimensional structures, adhesiveness to the Ti substrate by scratch tests, and durability of the CA films in buffered solution with different pH values. To perform in vitro biocompatibility tests, we conducted immersion experiments in simulated body fluid (SBF) with two different placements of samples, horizontal and vertical. Bone responses were evaluated after the implantation of an ASC-coated Ti disc in the femoral bone of rats. The bonding ability of ASC-coated Ti to bone was examined by push-in tests. The null hypothesis is that characteristics of CA coating are influenced by the conditions of the ASC method and that CA coating to Ti by the ASC method enhances bone formation and is applicable as a new coating technology for dental implants.

## 2. Results

### 2.1. Morphology and Surface Roughness

**Figure 1:**
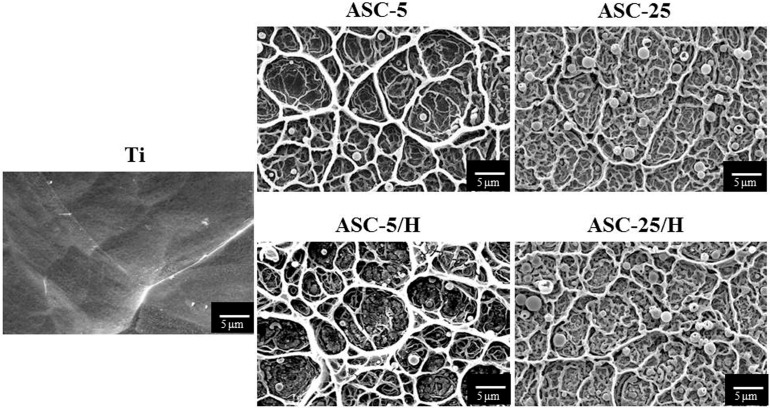
Scanning electron microscopy images of surface morphologies of the Ti and CA-coated specimens.

SEM (scanning electron microscope) pictures of the surfaces of the Ti substrate and four types of CA coatings films prepared by the ASC method revealed CA coatings covering the entire Ti surface (Figure 1). The network structure was approximately 10–15 μm and was observed across all four coating surfaces. Spherical particles with diameters of approximately 0.8–1.4 μm were present on the CA coating surface, with more spherical particles identified in ASC-25 and ASC-25/H in comparison to ASC-5 and ASC-5/H. Borders on the network structures were thicker in ASC-5 and ASC-5/H than in ASC-25 and ASC-25/H. The heat treatment produced no distinct differences in the surface morphology.

**Figure 2:**
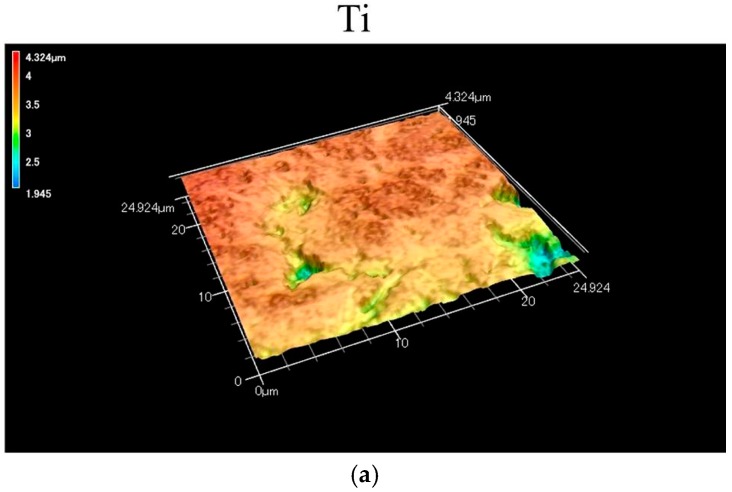

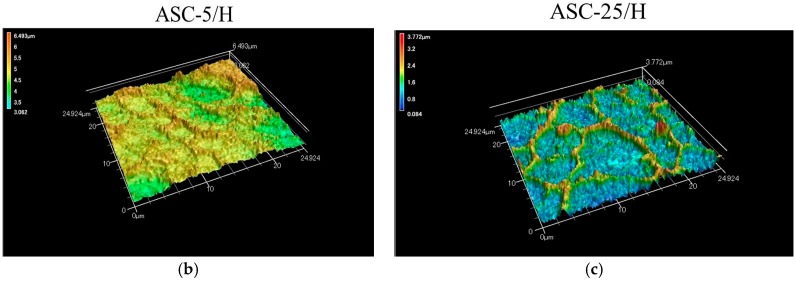
Three-dimensional images of the Ti and CA-coated specimens using a shape analyzer laser microscope: (**a**) Ti, (**b**) ASC-5/H, (**c**) ASC-25/H.

Laser microscopy of CA coatings revealed the three-dimensional surfaces of the heat-treated specimens (ASC-5/H and ASC-25/H), with ASC-25/H showing a clearer network structure (Figure 2). In addition, there were significant differences in both surface roughness parameters Sa (three-dimensional arithmetic height) and Sdr (surface deployment area rate) surface deployment area rate) among three specimens (*p* < 0.05; Table 1). ASC-25/H showed the greatest roughness (*p* < 0.05), and the Sa and Sdr values for ASC-25/H were approximately 1.5 and 4 times greater than that for ASC-5/H, respectively.

### 2.2. Adhesiveness of CA Coating

**Figure 3:**
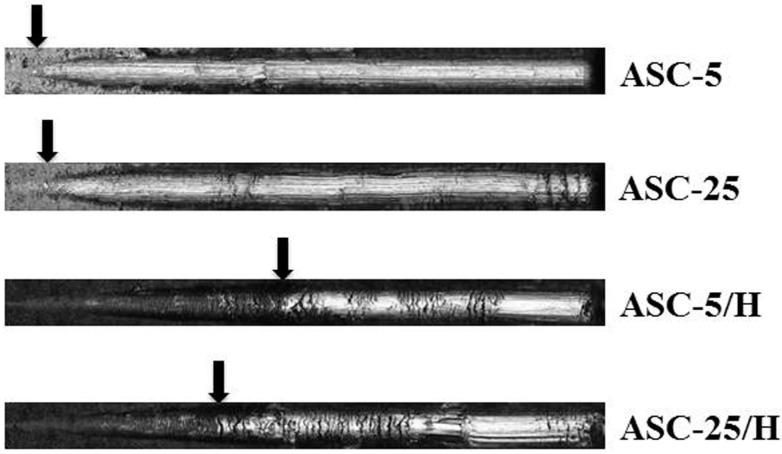
Panoramic images of the scratch trace, and at the first crack in the coating of representative samples. Arrows indicate the first crack in the coating. Lc values were obtained from these points.

Heat treatment produced significantly higher Lc (critical load) (values (*p* < 0.05) in the scratch test (Table 2). No significant differences in Lc values existed between ASC-5 and ASC-25, nor between ASC-5/H and ASC-25/H (*p* > 0.05). Moreover, there was a clearer break down of the coating film at the position of first crack for ASC-5 and ASC-25 in the panoramic images (Figure 3).

### 2.3. Durability

**Figure 4:**
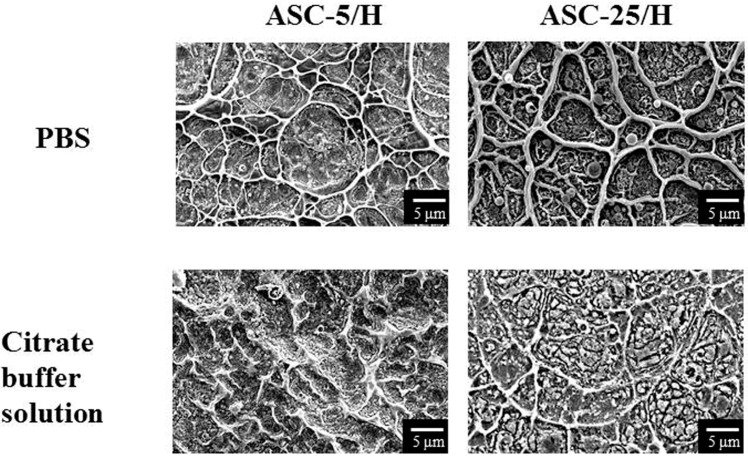
Scanning electron microscopy images of the surface morphology of CA films after immersion in phosphate buffered saline or citric acid buffer solution for one week.

After immersion in PBS (phosphate-buffered saline), there were no distinct differences in surface morphology for ASC-5/H and ASC-25/H, as detected by SEM (Figure 4). However, there was a slight dissolution in both ASC-5/H and ASC-25/H after immersion in citrate-buffered solution (Figure 4). Specifically, the dissolution of the protruding portion was distinct, and the borders of the network structures became unclear. Greater dissolution was observed in ASC-5/H than in ASC-25/H.

### 2.4. SBF Immersion

**Figure 5:**
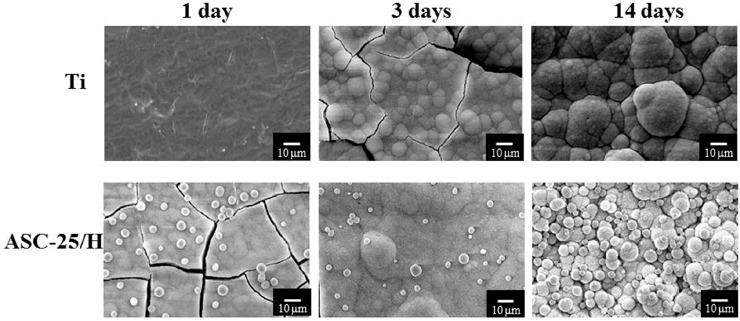
Scanning electron microscopy images of the surface appearance of Ti and heated aqueous spray coating samples after horizontal immersion in Hanks’ balanced salt solution.

**Figure 6:**
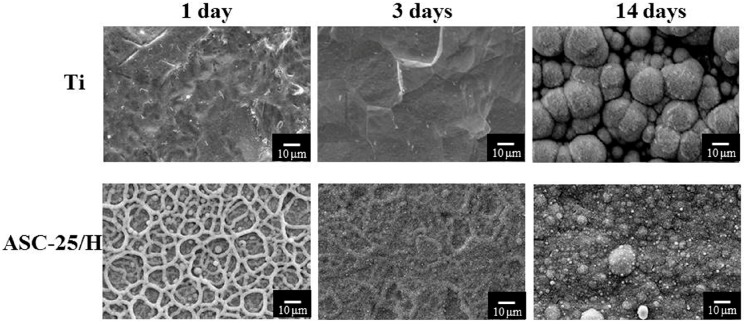
Scanning electron microscopy images of the surface appearance of Ti and heated aqueous spray coating sample after vertical immersion in Hanks’ balanced salt solution.

Clear differences were recognized in the crystal formation between Ti and ASC-25/H (Figure 5 and Figure 6). For horizontal placements, there were no crystals on Ti one day after immersion in SBF. However, ASC-25/H showed that network structures were completely covered by the crystals. Three days after immersion, crystals formed on Ti and ASC-25/H. Fourteen days after immersion, increased growth of spherical crystals was observed in ASC-25/H.

Vertical placement showed even greater indication of the differences in crystal formation between Ti and ASC-25/H. For Ti, no crystals were identified even three days after immersion (Figure 6). Conversely, crystal formation was observed in the network structure of ASC-25/H one day following immersion. Three days after immersion, the whole surface was nearly covered with crystals, and the network structure was barely recognizable. Fourteen days after immersion, both Ti and ASC-25/H showed crystal formation on their surfaces. However, although the entire Ti surface was not completely covered by the crystals, the surface of ASC-25/H was completely covered by the crystals. Therefore, a substantial difference in crystal growth behaviors was detected between horizontal and vertical placements.

**Figure 7:**
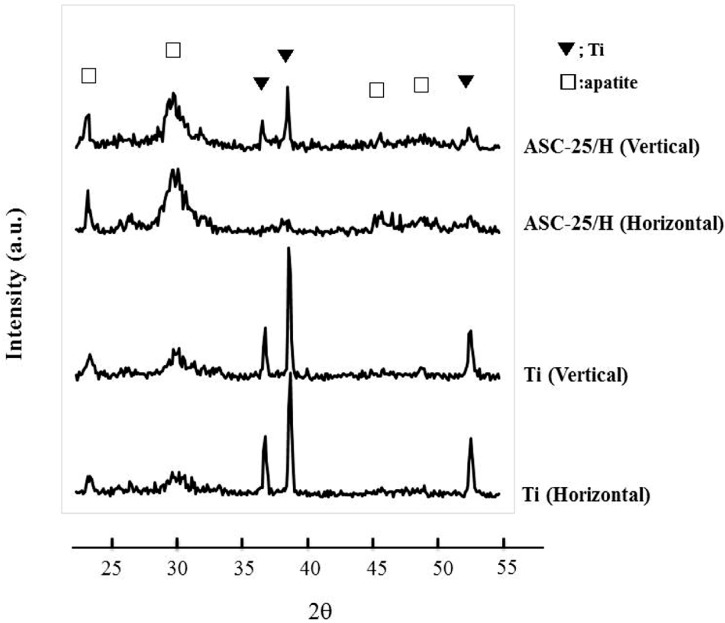
X-ray diffraction patterns of the precipitated crystals after immersion in Hanks’ balanced salt solution for 14 days.

**Figure 8:**
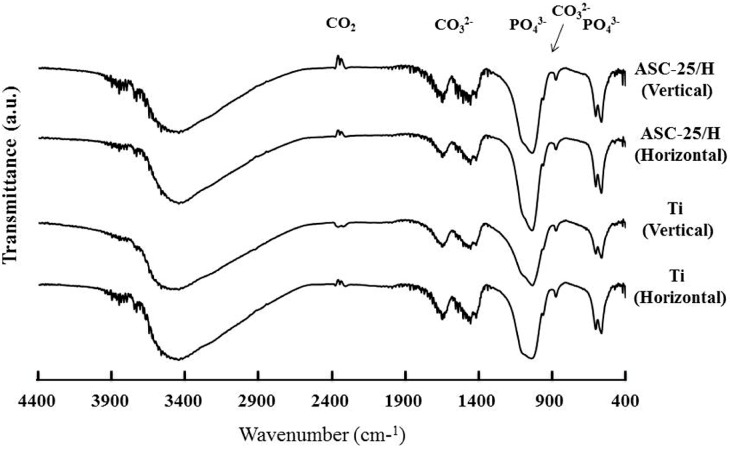
Fourier transform infrared spectra of the precipitated crystals after immersion in Hanks’ balanced salt solution for 14 days.

XRD (X-ray diffraction) patterns of the precipitated crystals 14 days after immersion revealed peaks derived from apatite structures at around 26.0°, 32.0°, 46.5°, and 49.5° (Figure 7). The FT-IR (Fourier transform infrared) spectra of the precipitated crystals 14 days after immersion revealed peaks derived from phosphate groups that were detected at approximately 550–600 cm^−1^ and 900–1200 cm^−1^, as well as peaks from carbonyl groups detected at approximately 800–900 cm^−1^ and 1300–1600 cm^−1^ (Figure 8). Therefore, precipitated crystals were identified as CA.

### 2.5. Histological and Histomorphometrical Evaluation

Experimental animals remained in good health and no failure of the implants was observed during the test period. No clinical signs of inflammation or adverse tissue reactions were seen when animals were sacrificed.

**Figure 9:**
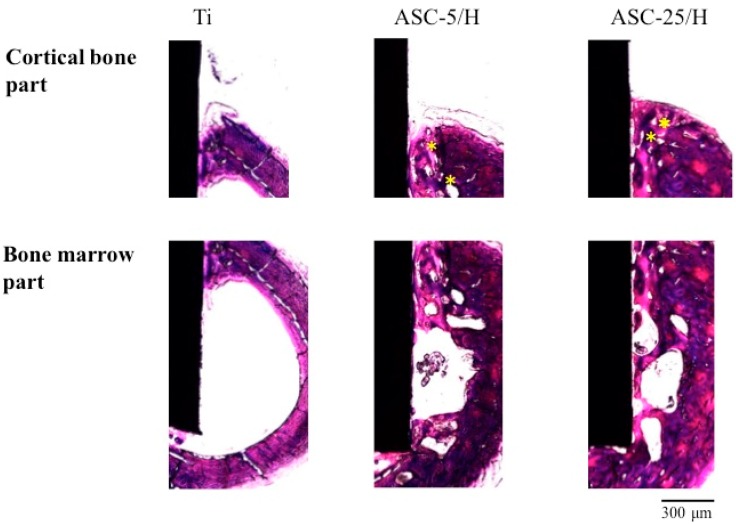
Histological appearances of Ti, ASC-5/H, and ASC-25/H implants two weeks after implantation. Asterisk: Haversian canal.

Differences in the histopathological appearances of cortical bone formation around the implants two weeks after implantation revealed new bone formation in all three implants (Figure 9). Frequently, no callus formation or other signs of wound healing was observed. The presence of original bone defects could not be identified. Haversian canals were observed in ASC-5/H and ASC-25/H, but not in Ti. The bone marrow demonstrated more distinct differences in new bone formation between Ti and CA-coated specimens. Greater amounts of new bone formation were observed for ASC-5/H and ASC-25/H than for Ti inside the bone marrow. Newly formed bone in bone marrow was trabecular bone and a part of new bone was formed close to the ASC-5/H and ASC-25/H.

**Figure 10:**
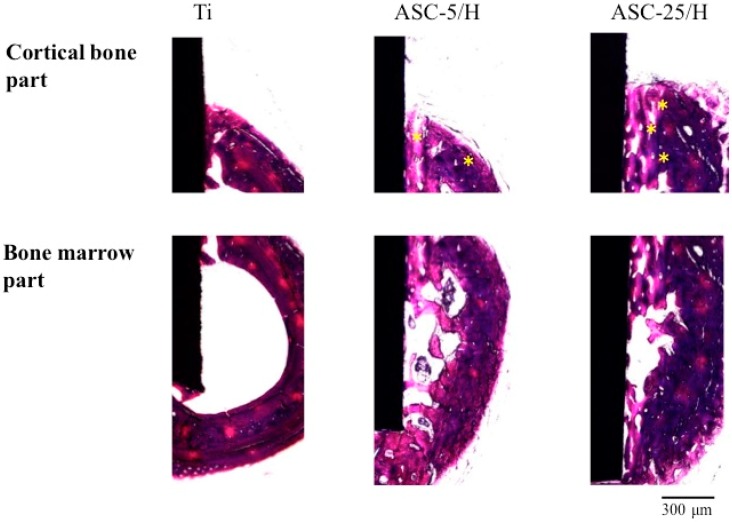
Histological appearances of Ti, ASC-5/H, and ASC-25/H implants four weeks after implantation. Asterisk: Haversian canal.

Four weeks after implantation, bone healing has proceeded and more mature bone formation was identified with the continuation of bone remodeling (Figure 10). Closer contact of bone formation toward ASC-5/H and ASC-25/H was identified relative to Ti in the cortical bone. Haversian canals were also observed in ASC-5/H and ASC-25/H, but not in Ti. In the bone marrow, ASC-5/H and ASC-25/H showed closer contact and more newly formed bone than Ti. Newly formed bone in bone marrow was trabecular bone, and more bone formation close to ASC-5/H and ASC-25/H was recognized compared with those observed at two weeks.

There were no significant differences in BIC (bone-to-implant contact) in the cortical bone among the three different implants two weeks after implantation (*p* > 0.05; Table 3). However, four weeks after implantation, ASC-5/H and ASC-25H showed significantly higher BIC than Ti, and BIC was the highest in ASC-5/H (*p* < 0.05). Indeed, BIC was significantly higher for ASC-5/H and ASC-25/H at four weeks post implantation than that at two weeks (*p* < 0.05).

In the bone marrow, ASC-25/H showed significantly higher BIC than Ti and ASC-5/H at two weeks after implantation. At four weeks after implantation, BIC of ASC-5/H and ASC-25/H was significantly higher than Ti (*p* < 0.05). No significant differences existed between ASC-5/H and ASC-25/H (*p* > 0.05). BICs four weeks post-implantation were significantly higher than those at two weeks for ASC-5/H and ASC-25/H (*p* < 0.05).

ASC-5/H and ASC-25H showed significantly higher BM (bone mass) than Ti (*p* < 0.05), and there were no significant differences between ASC-5/H and ASC-25H (*p* > 0.05; Table 4). At four weeks after implantation, significant differences were detected among the three different implants, with ASC-25/H showing the highest BM (*p* < 0.05). The BM of ASC-25/H increased significantly from two to four weeks (*p* < 0.05). No significant differences in BM were observed between two and four weeks for either Ti and ASC-5/H.

ASC-5/H and ASC-25/H showed significantly higher push-in loads than Ti (*p* < 0.05), and there were no significant differences between ASC-5/H and ASC-25H (*p* > 0.05; Table 5).

## 3. Discussion

In the present study, we characterized thin CA coatings produced using the ASC method, and their biocompatibility was evaluated using both in vitro SBF immersion experiments and in vivo animal implantation experiments. It revealed that characteristics of ASC coating were influenced by the conditions of the ASC method such as spraying amounts and heat treatment. Moreover, ASC coating can enhance bone formation. Therefore, the null hypothesis is accepted.

The ASC method is a new technique to deposit thin CA coatings onto Ti. The novelty of the ASC method is that it produces thin CA films with network structures. The size of the present Ti substrate is smaller than previous ones employed for animal implantation experiments and the current ASC method can produce network structures similar to a former report on smaller Ti substrates [28,29]. Briefly, a sprayed solution generates concentrated droplets in the mist while traveling from the nozzle to the plate, and a CA film deposits due to the collision of the concentrated droplets that follows water evaporation on the sheathed heater. Although the ESD method also can produce a similar network structure, the network size on the CA coating surface is 5–8 μm thinner with the ASC method [14,15]. In addition, the ASC method is unique in that it can produce rounded CA particles inside the network structure. The presence of small particles made for a rougher surface. Indeed, ASC-25/H had a rougher surface than ASC-5/H due to the greater amount of spray solution.

The adhesive nature of the CA coating was evaluated using scratch tests. The adhesiveness of the thin CA coating produced by the molecular precursor method was also evaluated using scratch tests [23,30]. A previous study for ASC coating evaluated the adhesiveness of only heat-treated CA coating specimens [28]. In the present study, it revealed that heat treatment improved the adhesion of CA coating to Ti. It is speculated that the improvement in the density of the CA coating film due to heat treatment is caused a higher bonding of the CA coating. A more apparent breakdown of the coating film, which was observed in ASC-5 and ASC-25, was due to the brittleness of the CA coating film. Heat treatment reduced this brittleness due to improvement in density. Non-heat-treated specimens have a risk of the CA coating peeling from the Ti substrate during storage or surgical operation. Therefore, we conclude that the heat treatment of CA coating is necessary for use in clinical application, and heat-treated specimens were used for durability, SBF immersion, and animal experiments in this study.

The durability of CA coatings was evaluated by immersion in two different pH buffer solutions. It is well known that the inflammation caused by drilling or other surgical procedures can produce acidic conditions in tissues that receive implanted materials. Therefore, citrate-buffered solution with a pH of 5.4 was used to simulate inflammatory conditions and revealed that acidic conditions accelerated, but did not completely cause, the dissolution of CA coatings. Due to the greater amounts of spraying on ASC-25/H, there was more CA coating remaining than on ASC-5/H. It is therefore predicted that CA-coated implants inserted into the drilled hole will result in partially dissolved living tissue. Although the mechanism that provides greater better bone formation is remains unclear, it is possible that the elution of calcium ions from dissolved CA film improves the local calcium concentration and helps to activate osteoblast formation.

As for the in vitro evaluation of biocompatibility, SBF immersion experiments were performed. In the present study, we used HBSS (Hanks’ balanced salt solution) as an SBF [31]. Hanawa et al. [31] reported that an apatite layer formed on a titanium surface after immersion in HBSS. A previous study reported that the difference in spray amounts had little influence on crystal precipitation on the CA-coated surface [29]. Therefore, we only used one heat-treated sample, ASC-25/H, for the immersion study. In addition to CA coating, we investigated whether horizontal or vertical placement of samples in HBSS influenced crystal formation. Although previous studies have generally used horizontal placement in HBSS solution, Suzuki et al. [32] reported that apatite formation and growth on Ti could be influenced by the placement and orientation of samples in SBF immersion experiments. They revealed that CA coating promoted crystal formation after immersion in SBF for both vertical and horizontal placements. Therefore, a better response of bone growth for CA-coated implants would be expected in vivo. However, it appeared that vertical placement led to greater crystal formation and growth than horizontal placement. It is presumed that in vivo mineral formation system would be more substantial, and the influence of the direction of the sample placement on mineralization behaviors should be studied further.

Many previous studies have revealed the ability for apatite formation in vitro, which has led to the common prediction that these materials also have in vivo bioactivity [33]. We used animal experiments to reveal that CA coating specimens ASC-5/H and ASC-25/H provided significantly greater amounts of BIC in cortical bone and bone marrow than Ti alone. ASC-5/H was more effective at increasing BIC than ASC-25/H. It is well known that rougher surfaces provide faster and more bone formation [34]. However, the surface roughness did not contribute to an increase of BIC in the present study. Mochizuki et al. [29] previously evaluated the attachment, proliferation, and differentiation of osteoblast-like cells on CA-coated Ti. They found that initial attachments of osteoblast-like cells increased due to CA coating and no difference was observed between ASC-5/H and ASC-25/H. On the contrary, cell differentiation was enhanced more on ASC-5/H than on ASC-25/H. They speculated that reduced border heights in the network structure of CA coating of ASC-5/H was preferred for the spreading of the osteoblast-like cells, and as a result, mineralization would be more accelerated with ASC-5/H. Therefore, higher BIC in the cortical part was obtained for ASC-5/H in the present animal experiments.

Albrektsson et al. [35] suggested that osseointegration corresponded to approximately 60% bone contact for titanium implants. The present BICs of the ASC-5/H and ASC-25/H were above the limit proposed by Albrestsson et al. [35], not only for cortical bone but also for bone marrow.

We inserted the implants in the cortical part of the rat femur. Cortical bone is known to be denser and stiffer than trabecula bone. The elastic modulus of cortical bone was previously shown to be higher that of trabecular bone [36]. The implant site between cortical and trabecular bone has been shown to influence the bone response to implants [37]. Hayakawa et al. [38] reported that cortical and trabecular bone exhibited different bone responses towards apatite-coated titanium implants. Siebers et al. [39] reported that the application of an ESD CA coating resulted in more bone contact compared with Ti implants after the implantation into the trabecular bone of the femoral condyle of the goat. The bone response after the implantation into the trabecular bone should be further evaluated.

New bone formation in the bone marrow generated novel insights for CA coating. In this case, ASC-25/H, which has a higher border height in the network structure than ASC-5/H, enhanced more new bone formation in the bone marrow. Bone marrow is known to be rich in hematopoietic stem cells. Therefore, it is speculated that the ASC coating stimulated the activity of hematopoietic stem cells in the bone marrow. Specifically, ASC-25/H will release more calcium and increase the local concentration of calcium. As a result, ASC-25/H will produce more new bone formation in the bone marrow. Other factors, including mechanical factors, may influence bone formation. Further studies should be conducted to elucidate the mechanism of new bone formation in bone marrow.

A push-in test was performed to evaluate the bonding between the implant and surrounding bone. Both CA coating implants produced tighter bonding to bone than Ti. Surface roughness did not influence the values in push-in tests. Lin et al. [40] also reported that surface modification with hydroxyapatite nanoparticles increased the push-in values two weeks after implantation into the femur of rats. In the present study, we only monitored the peaks at the load–displacement curve. Detailed observation of the failure surface after the push-in test will be necessary to analyze the details of bonding behaviors.

ASC methods were employed to disc-shaped or rectangular-plate Ti in the present study. To apply the ASC method for dental clinics, CA coatings for cylindrical or screw-shaped Ti implants must be developed. For that purpose, a rotating jig with small a heater is being developed. Cylindrical or screw-type specimens have been set on the rotating jig, and various conditions, such as rotating speed, heating, and spraying methods, are now under investigation.

## 4. Materials and Methods

### 4.1. Experiment Specimens

We prepared two shapes of commercially pure Ti in this study: Disc-shaped Ti specimens (Diameter 1.5 mm, thickness: 1.0 mm, JIS 2 type, 99.9% mass, Furuuchi Chemical Corp., Tokyo, Japan) and rectangular plate specimens (2 × 1.5 × 0.5 mm, JIS2 type, 99.9% mass, Furuuchi Chemical Corp., Tokyo, Japan). Disc-shaped Ti specimens were used to characterize CA thin films in SBF immersion experiments, and rectangular plate specimens were used for animal experiments. Emery paper, ranging in grit size from #100 to #1200, was used in succession to polish the Ti surface under running water. Afterwards, polished specimens were washed with acetone for 15 min and deionized water for 10 min under ultrasonication.

### 4.2. Preparation of ASC Solution and Spray Coating

The ASC solution was prepared according to previous reports [28,29]. Briefly, calcium hydroxide (Wako Pure Chemical Industries, Osaka, Japan) was suspended in deionized water, and then CO_2_ gas was introduced into the suspended solution by ultrasonication until a clear solution was obtained. Phosphoric acid (Wako Pure Chemical Industries, Osaka, Japan) was added into the clear solution with a Ca/P ratio of 1.67. The final clear solution was stored in a refrigerator until use.

**Figure 11:**
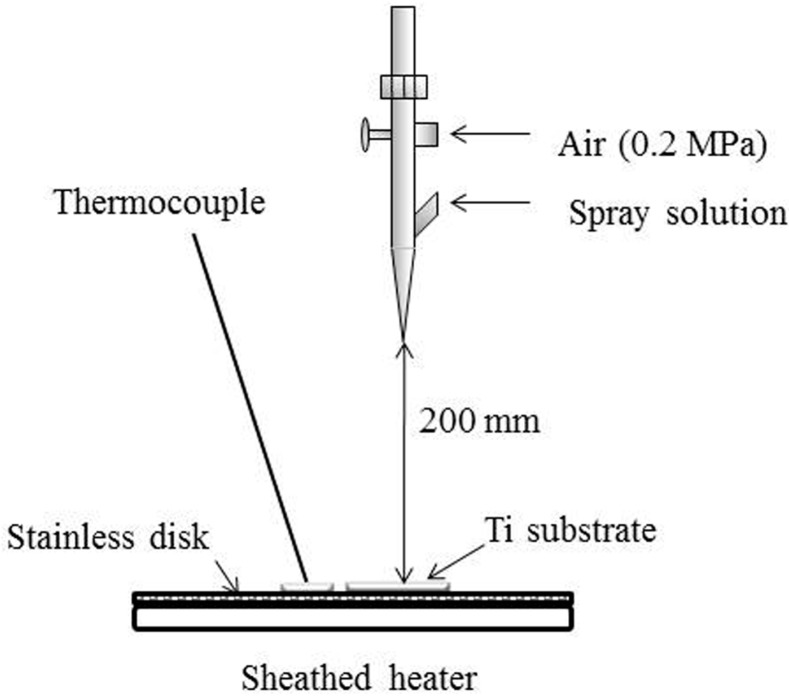
A schematic representation of the apparatus for aqueous spray coating on titanium.

Figure 11 shows a schematic drawing of the CA coating using the ASC method [28,29]. An aqueous solution was sprayed onto a disc-shaped or rectangular plate Ti through the nozzle of an air brush (HP-SAR, ANEST IWATA, Kanagawa, Japan). A sheathed heater was used for controlling the Ti substrate at 40 °C. The Ti substrate was placed in the center of the stainless-steel plate at a perpendicular distance of 200 mm using the spray nozzle. Air pressure during spraying was set at 0.2 MPa and spraying speed at 5 mL/min. Two as-sprayed samples, ASC-5 or ASC-25, were obtained by spraying 5 mL or 25 mL of the ASC solution from the spray nozzle onto the Ti substrate, respectively. The sprayed samples then were heated at 600 °C for 10 min under Ar gas flowing at a rate of 0.5 mL/min using a high-speed desktop electric heating furnace (EPKPO12-K, Isuzu Seisakusho, Tokyo, Japan). Hereafter, the heated ASC-5 and ASC-25 samples are abbreviated as ASC-5/H and ASC-25/H.

### 4.3. Surface Morphologies and Surface Roughness of CA Film

The surface morphology of the CA film deposited on the Ti substrate was observed under a scanning electron microscope (SEM; JSM-5600LV, JOEL Ltd., Tokyo, Japan) at an accelerating voltage of 15 kV after sputter coating with Au using an ion coater (QUICK COATER SC-701, Sanyu Electron, Tokyo, Japan). Three-dimensional observations of surface morphology were performed by a shape analyzer laser microscope (VK-X250, KEYENCE, Osaka, Japan). Images were acquired in three-dimensional ranges of decreasing size of 25 × 25 μm^2^. Two surface parameters, three-dimensional arithmetic height (Sa) and surface deployment area rate (Sdr), were obtained.

### 4.4. Adhesiveness of CA Films

The adhesiveness of the CA thin film to the Ti surface was evaluated using a diamond-stylus scratch method (Nano Scratch Tester, Anton Paar, Graz, Austria). A diamond stylus (rockwell type, tip radius: 200 μm) was moved over each specimen surface under a linearly increasing load until failure occurred. The different scratch test parameters varied as follows: The begin to end load was 1 to 10 N, and the speed of the moving stage was 5 mm/min (length: 4.95 mm). The critical load (Lc) was defined at the first crack in the coating. Scratch tests were performed in three places for each of four specimens, ASC-5, ASC-25, ASC-5H, and ASC-25H, and Lc values were calculated. Measurements were performed thrice for each specimen.

### 4.5. Durability of CA Coating

Following heat treatment, CA-coated discs (ASC-5/H or ASC-25/H) were immersed in 20 mL of phosphate-buffered saline (PBS) solution with pH = 7.4 or citrate-buffered solution with a pH = 5.4 in a polypropylene bottle for one week. During immersion, solutions were changed every day. After immersion, the specimens were dried in a desiccator. The change in morphology of the CA films was observed by SEM at an accelerating voltage of 15 kV after sputter coating with Au.

### 4.6. SBF Immersion

Hanks’ balanced salt solution (HBSS), without organic species, was employed as an SBF [31]. Ti and ASC-25/H discs were immersed in 20 mL of HBSS with an adjusted pH of 7.4 at 37 °C in a polypropylene bottle. Each specimen was placed either horizontally or vertically in HBSS for immersion experiments, as previously described [32]. For horizontal placement, discs were placed directly at the base of the bottle. For vertical placement, the discs were hung from a nylon wire. The nylon wire was attached to each disc with a plastic clip. To ensure that the discs were constantly exposed to fresh medium, the medium and container were changed daily. At one, three, and 14 days after immersion, the discs were rinsed with double distilled water and immediately dried in a desiccator. The surface appearance of each disc after immersion in HBSS was observed using SEM at an accelerating voltage of 15 kV after sputter coating with Au.

The crystallographic structure of the deposited crystals on CA-coated specimens was analyzed after 14 days of immersion using X-ray diffraction (XRD, θ–2θ, MXP-18 AHF22; Bruker AXS, Kanagawa, Japan), which yielded an X-ray source of Cu-Kα, a 50-kV voltage, and a 50-mA current. Fourier transform infrared (FT-IR) spectroscopy (FT/IR-600, JASCO, Tokyo, Japan) was also performed using the KBr method to analyze the results.

### 4.7. Animal Experimentation

The animal experiment was approved by the animal experiment committee guidelines of the Tsurumi University School of Dental Medicine (approval No. 27A055, No. 28A059, and No. 28A067). A total of 36 six-week-old male Wistar rats weighing 180–200 g were used. Twenty-four rats were used for histological and histomorphometrical evaluation, and 12 rats were used for push-in tests. All animals were housed in a temperature-controlled room with a 12 h alternating light–dark cycle and were provided water and food (standard rat-chew) ad libitum during the experimental period.

Each animal received one rectangular plate implant. A total of 36 implants were inserted. In particular, four Ti, four ASC-5/H, and four ASC-25/H were implanted for two weeks, and four Ti, four ASC-5/H, and four ASC-25/H were implanted for four weeks. These implants were used for histological and histomorphometrical evaluation. In addition, four Ti, four ASC-5/H, and four ASC-25/H plates were implanted for two weeks and used in push-in tests. Before all animal experiments, all plate implants were sterilized using ethylene oxide gas.

Surgical interventions were conducted under general anesthesia by an intraperitoneal injection of ketamine hydrochloride (0.8 mg/kg) and medetomidine hydrochloride (0.4 mg/kg). Local anesthesia was induced with lidocaine hydrochloride (1.8 mL) and epinephrine bitartrate (0.045 mg) (ORA Injection, Showa Yakuhin Kako, Tokyo, Japan). Surgical procedures were performed in accordance with the methods reported by Suzuki et al. [41]. The right hind limb was shaved after sterilization with ethanol. A longitudinal incision was made on the distal surface of the right hind limb to expose the femur. A bone defect was generated with a very gentle surgical technique with a continuous internal cooling using physiological saline solution. A cortical bone defect measuring 0.8 × 2.0 mm was created through the cortex and medulla at a length of approximately 15 mm from the head of the femur using a carbide bur (#700, Shofu, Kyoto, Japan) at a low rotational speed.

After the insertion of the implants into the bone defects by press-fitting, muscle tissue and skin were closed in separate layers by the single-knot technique using nonabsorbable sutures (BioFit-D 5-0, Washiesu Medical Corp., Tokyo, Japan; Nylon 4-0, Mani, Utsunomiya, Japan). To reduce the risk of perioperative infection, a prophylactic antibiotic equivalent to latamoxef sodium (0.01 mg/kg Shiomalin, Shionogi & Co., Osaka, Japan) was administered postoperatively by subcutaneous injection. The animals were sacrificed at two and four weeks after implantation. The femurs were harvested and fixed in 10% neutralized formalin for histological observation.

### 4.8. Histological and Histomorphometrical Evaluation

The fixed specimens were dehydrated in a graded series of ethanol and embedded into methylmethacrylate. After polymerization, non-decalcified thin sections were prepared using a cutting-grinding technique (EXAKT-Cutting Grinding System, BS-300CP band system and 400 CS microgrinding system, EXAKT Appratebau, Norderstedt, Germany) [42]. Sections of approximately 50–70 µm were made in a transverse direction perpendicular to the long axis of the implants. Undecalcified sections were stained with methylene blue and basic fuchsin and were evaluated using a light microscope (BX51, Olympus Corp., Tokyo, Japan, magnification 200×).

**Figure 12:**
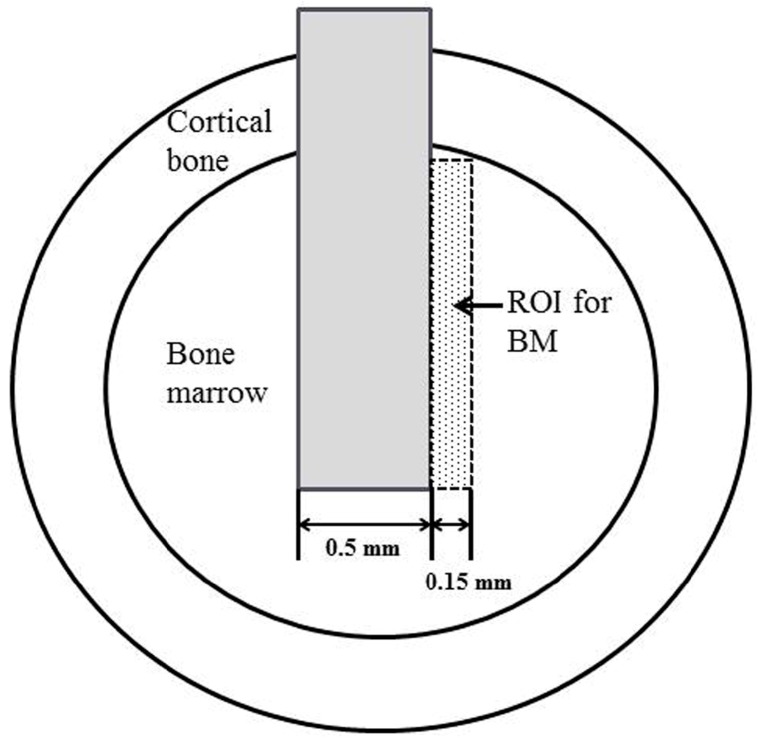
A schematic drawing of the region of interest (ROI) for quantitative analysis of histomorphometrical measurements, bone-to-implant contact (BIC), and bone mass (BM).

We estimated the percentage of bone-to-implant contact (BIC) and bone mass (BM) around the implants using histomorphometrical and image analysis (WinROOF, Visual System Division, Mitani, Tokyo, Japan). BIC was calculated as the percentage of the length of bone-implant contact in the cortical bone and bone marrow. BM was defined as the percentage of newly formed bone within the region of interest in the bone marrow, which is illustrated in Figure 12.

BIC rate was calculated as the percentage of the length of bone-to-implant contact in the cortical bone and bone marrow. BM rate was defined as the percentage of newly formed bone within the region of interest (ROI) and is indicated with a dotted line.

### 4.9. Implant Push-In Test

An implant push-in test was conducted to evaluate the bonding between the implant body and surrounding bone [40]. Two weeks after implantation, the femurs of rats were extracted and the long axis of the embedded implant plate was placed vertical to the base using wax. The implant was pushed with a custom-made pushing rod (diameter, 0.8 mm) at a cross head speed of 1 mm/min using a universal testing machine (Shimadzu Autograph AG-IS 20 kN, Shimadzu, Kyoto, Japan). The applied load and displacement of the implant were monitored. The push-in value was determined by measuring at the peak of the load-displacement curve.

### 4.10. Statistical Analysis

Data for surface roughness, scratch tests, BIC, and BM of different implants were analyzed using one-way analysis of variance and the post hoc Tukey’s test for multiple comparisons among means. We used an unpaired *t*-test to determine if BIC and BM differed between implant times (two-week or four-week treatments). Statistical analyses were conducted with Origin Pro 9.0 J (OriginLab Corp., Northampton, MA, USA). *p* values of less than 0.05 were considered significant, and data were expressed as the mean ± standard deviation.

## 5. Conclusions

In the present study, thin CA films were deposited onto Ti using the ASC method. Heat treatment improved the bonding of the CA thin film to Ti. Acidic conditions increased the dissolution of the CA thin film. Crystal formation after immersion in SBF progressed on CA-coated specimens. The direction of sample placement further influenced crystal formation and growth. Animal implantation experiments revealed that ASC-5/H showed a greater BIC than AS-25/H in the cortical part four weeks after implantation, but new bone formation in the bone marrow was enhanced more with ASC-25/H than that with ASC-5/H. The bonding of CA coating to titanium is clinically acceptable. Therefore, we conclude that CA coating on Ti by the ASC method could be used for clinical applications, including dentistry.

## Figures and Tables

**Table 1:** Surface roughness (Sa and Sdr) of specimens.

Specimen	Sa (μm)	Sdr (μm)
Ti	0.17 (0.01) ^a^	0.17 (0.09) ^d^
ASC-5/H	0.31 (0.04) ^b^	4.34 (0.35) ^e^
ASC-25/H	0.46 (0.06) ^c^	16.76 (2.60) ^f^

Values in brackets are SD (standard deviation). Different superscript letters indicate a significant difference (*p* < 0.05).

**Table 2:** Lc (critical load) values obtained by scratch test.

Specimen	Lc Value (mN)
ASC-5	4.1 (0.5) ^a^
ASC-25	6.8 (0.3) ^a^
ASC-5/H	36.1 (10.5) ^b^
ASC-25/H	23.8 (4.7) ^b^

Values in brackets are SD. Different superscript letters indicate a significant difference (*p* < 0.05).

**Table 3:** Measured BIC (bone-to-implant contact) in cortical bone and bone marrow part of implant specimens.

Specimen	Cortical Bone Part		Bone Marrow Part	
	Two Weeks	Four Weeks	Two Weeks	Four Weeks
Ti	29.7 (14.8) ^a,A^	46.1 (7.6) ^b,A^	22.6 (6.9) ^e,F^	27.6 (14.8) ^g,F^
ASC-5/H	41.4 (11.7) ^a,B^	78.7 (4.8) ^c,C^	34.9 (12.3) ^e,f,G^	72.3 (18.6) ^h,H^
ASC-25/H	46.1 (10.0) ^a,D^	64.1 (8.2) ^d,E^	46.5 (9.4) ^f,I^	63.8 (10.2) ^h,J^

Values in brackets are SD. Different superscript letters indicate a significant difference (*p* < 0.05). Small letters indicate a difference between implant materials with the same implantation period. Large letters indicate a difference between two and four weeks with the same implant material.

**Table 4:** Measured BM (bone mass) in bone marrow part of implant specimens.

Specimen	Two Weeks	Four Weeks
Ti	21.0 (1.6) ^a,A^	21.1 (6.5) ^c,A^
ASC-5/H	31.8 (4.0) ^b,B^	41.6 (11.2) ^d,B^
ASC-25/H	35.3 (7.6) ^b,C^	62.4 (4.3) ^e,D^

Values in brackets are SD. Different superscript letters indicate a significant difference (*p* < 0.05). Small letters indicate a difference between implant materials with the same implantation period. Large letters indicate a difference between two and four weeks with the same implant material.

**Table 5:** Measured push-in loads of implant specimens.

Specimen	Load (N)
Ti	3.3 (0.2) ^a^
ASC-5/H	23.4 (5.5) ^b^
ASC-25/H	20.1 (0.7) ^b^

Values in brackets are SD. Different superscript letters indicate a significant difference (*p* < 0.05).
